# Agarose-based structured optical fibre

**DOI:** 10.1038/s41598-020-64103-3

**Published:** 2020-04-27

**Authors:** Eric Fujiwara, Thiago D. Cabral, Miko Sato, Hiromasa Oku, Cristiano M. B. Cordeiro

**Affiliations:** 1 Laboratory of Photonic Materials and Devices, School of Mechanical Engineering, University of Campinas, Campinas, 13083-860 Brazil; 2 “Gleb Wataghin” Institute of Physics, University of Campinas, Campinas, 13083-859 Brazil; 3 Graduate School of Science and Technology, Gunma University, Kiryu, 376-8515 Japan

**Keywords:** Applied optics, Other photonics

## Abstract

Biocompatible and resorbable optical fibres emerge as promising technologies for *in vivo* applications like imaging, light delivery for phototherapy and optogenetics, and localised drug-delivery, as well as for biochemical sensing, wherein the probe can be implanted and then completely absorbed by the organism. Biodegradable waveguides based on glasses, hydrogels, and silk have been reported, but most of these devices rely on complex fabrication procedures. In this sense, this paper proposes a novel structured optical fibre made of agarose, a transparent, edible material used in culture media and tissue engineering. The fibre is obtained by pouring food-grade agar into a mould with stacked rods, forming a solid core surrounded by air holes in which the refractive index and fibre geometry can be tailored by choosing the agarose solution composition and mould design, respectively. Besides exhibiting practical transmittance at 633 nm in relation to other hydrogel waveguides, the fibre is also validated for chemical sensing either by detecting volume changes due to agar swelling/dehydration or modulating the transmitted light by inserting fluids into the air holes. Therefore, the proposed agarose-based structured optical fibre is an easy-to-fabricate, versatile technology with possible applications for medical imaging and *in vivo* biochemical sensing.

## Introduction

Biocompatible and biodegradable optical fibres are emerging, breakthrough technologies for enabling the assessment and manipulation of biological systems by their interaction with light. Such devices are eligible as implantable probes for *in vivo* measurements^1,2^ and endoscopes for medical imaging^3^, being completely absorbed by the organism after their use. Another promising application is the monitoring of biochemical variables, wherein the waveguide can be used both as the sensing probe and encapsulation medium for microorganisms^4^. Moreover, localized and controlled light incidence is suitable for optical actuation purposes, including drug-delivery, laser-assisted surgeries^5^, photothermal therapy^6^, and neuronal stimulation through optogenetics^7^.

Nowadays, several types of biodegradable waveguides have been reported. Calcium phosphate, a bioresorbable glass, was used to fabricate optical fibres with relatively low attenuation and single-mode characteristics at the near-infrared^2^. This material is also suitable to inscribe Bragg gratings for wavelength selection purposes^8^, but applicability in invasive procedures is limited since fractured glass fibres can cause injuries due to sharp edges^9^. As to polymer devices, a citrate-based fibre with improved optical and mechanical properties was developed and successfully tested on imaging and *in vivo* analyses^3^. In another work, poly(L-lactic acid)-based fibres obtained through preform drawing were conceived as implantable waveguides, exhibiting low attenuation coefficient and excellent biocompatibility^10,11^. Remarkable results were also achieved by microstructured dual-core fibres made of cellulose butyrate. By processing stacked tubes in a drawing tower, cylindrical biodegradable waveguides can be fabricated with solid or hollow cores, which is desirable for potential applications in chemical measurements and microfluidics, whereas the optical properties and sensing capabilities can be easily tailored by adjusting the fibre geometry^5,12^. Ultimately, silk fibroin from silkworms or spiders can be processed into planar waveguides and optical fibres through soft lithography and nanoimprinting, exhibiting good stability, biochemical affinity, and reliable optical characteristics for integrated photonics and optoelectronics^9,13–15^.

Despite recent advances in biocompatible glass and polymer fibres, light waveguides made of hydrogels present remarkable characteristics in terms of tailoring optical and mechanical properties of the material by controlling the polymer/water content, yielding transparency, flexibility, and stability^16,17^. Fabrication routes are usually simpler than other fibre types^2,10,14^, and hydrogels are also suitable for functionalization by embedding biomolecules in the material pores as well as for cell encapsulation^16^. Ultimately, intrinsic sensing capabilities can be achieved by taking advantage of the hydrogels volume change in response to temperature, pH, and solute concentration of surrounding media^16,17^.

For instance, slab waveguides made of poly(ethylene glycol) (PEG) coupled to standard optical fibres were developed for detecting heavy metal ions *in vivo*, either by encapsulating cells or embedding fluorescent nanoparticles^7,18^. Hydrogel-based fibres were also conceived with PEG core and alginate cladding structure^1,6,19^, wherein the monomer solution is injected in a tube and cured using UV light to form the fibre core. Then, the material is removed from the mould and coated by dipping the fibre in a sodium alginate and calcium chloride solution, yielding low relative transmission losses, and the capability to assess blood oxygen saturation^1^ and glucose concentration^19^. Another approach is comprised of an alginate-acrylamide multimode fibre by adjusting the numerical aperture according to the acrylamide concentration, presenting sensitivity to refractive index and strain^20^.

Besides the aforementioned hydrogels, agarose exhibits excellent biocompatibility and provides excellent physical and biochemical conditions for cell encapsulation and nutrients permeation^4,21^. Agarose also presents low-cost, food-grade characteristics, thermo-reversible gelation, and high transparency in the visible range, whereas the refractive index can be increased by adding sugar to the hydrogel solution^21,22^. In a previous work, a gelatine core, agarose cladding planar waveguide was fabricated by spin coating to obtain devices with core thickness ranging from 2 µm to 2 mm, providing practical transmittance in the visible spectrum over 50 mm length^23^. Another approach comprised of a 130 × 130 µm² cross-section area rib waveguide made of agarose through soft lithography method exhibited 13 dB/cm transmission loss and the ability to encapsulate living cells^4^. However, there is no mention of agar-based optical fibres in the literature to the best of our knowledge.

In this context, this paper reports a novel structured optical fibre made of food-grade agarose. This is the first realization of an agar-based fibre in contrast to the previous planar and rib waveguides^4,23^, comprising a straightforward fabrication method to produce devices eligible for *in vivo* imaging and light delivery since agar is biocompatible and edible. Moreover, this is the first structured hydrogel fibre reported in the literature, making it sensitive to fluids surrounding the cladding or inserted into the air holes. Besides the characterization of optical properties, the fibre was tested on the assessment of fluids through speckle field analysis^24^ using a simple interrogation setup. Such features make the proposed device attractive to chemical and biological sensing applications once the optical characteristics can be readily tailored by adjusting the fibre geometry and agar composition during its fabrication.

## Results

### Fibre fabrication and characterization

**Figure 1:**
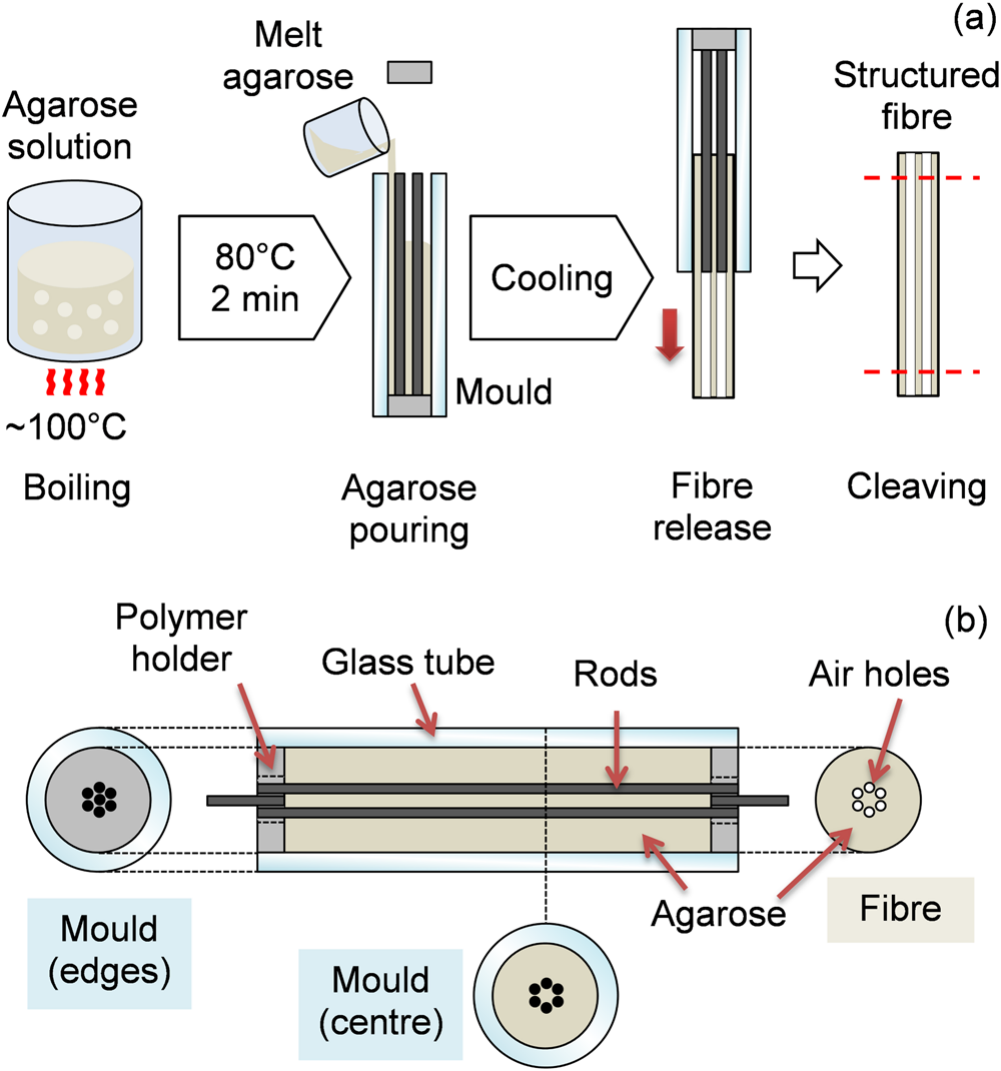
(**a**) Fabrication of structured agarose fibre: boiled agar solution is poured into the mould, and the fibre is released after solidification in a refrigerator. (**b**) Detail of the fibre inside the mould with cross-section views of the mould edges and centre, as well as the fibre end face: the glass tube supports the fibre structure whereas air holes are formed by the rods.

The fabrication method (Fig. 1) consists of pouring food-grade agarose solution into a glass mould (inner diameter of 3 mm) with six internal rods (diameter of 0.5 mm). The rods are arranged in symmetrical fashion and fixed to the external tube using polymer holders, whereas central rods are connected to the extremities for supporting the structure and create the fibre core. After cooling, the rods are removed to form air holes, and the solidified waveguide is released from the mould and cleaved.

**Figure 2:**
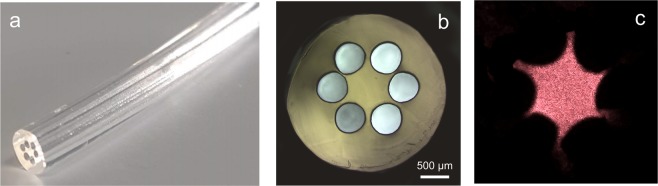
(**a**) Agarose-based structured optical fibre: (**b**) cross-section view of the end-face and (**c**) output speckle field of the core-guided modes. The fibre has 60 mm length, diameters of 0.64 mm, 2.5 mm, and 0.5 mm for core, cladding, and holes respectively, and bridges of ~0.08 mm width.

A multimode optical fibre made of 2% w/v agarose is shown in Fig. 2(a,b). To demonstrate the guiding capabilities, light from a HeNe laser illuminates the fibre core, producing the far-field speckle-like pattern observed in Fig. 2(c), which is related to the interference between several propagating modes typical of multimode optical fibres^24^. Likewise microstructured glass fibres^25^, light is confined to the core due to the refractive indices difference between air holes and fibre material. Cladding and holes follow the mould dimensions (~2.5 mm and ~0.5 mm, respectively) whereas the core (~0.64 mm diameter) is supported by ~0.08 mm width agar bridges, suggesting that the fibre size could be reduced by choosing the mould geometry accordingly.

**Figure 3:**
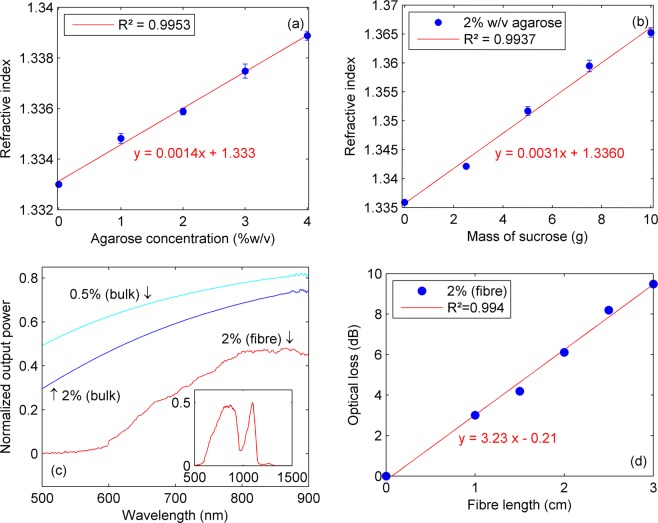
Optical characterization: (**a**) refractive index of bulk agarose samples; (**b**) refractive index as a function of sucrose addition in a 2% w/v agarose samples; (**c**) output power spectra for agarose bulk and fibre samples, the inset shows the output spectrum of a 2% w/v agarose fibre; and (**d**) fibre optical loss at 633 nm obtained by the cut-back method.

The optical characteristics of structured fibre depend on the composition of agar solution. For instance, Figure 3(a,b) shows the effect of concentration on the refractive index (RI) of bulk agarose samples. The experiments were conducted for pure agar (0 to 4% w/v) as well as for sucrose addition to 2% w/v agarose solutions. Refractive index increases linearly with concentration, yielding ~1.4 × 10^−3^ and ~3.1 × 10^−3^ RIU/g (RI unit per gram) enhancement for agarose and sucrose, respectively, making it feasible to adjust the fibre light guiding characteristics.

Output light spectra of bulk samples were measured in a spectrophotometer, whereas the fibres were excited with supercontinuum source and characterized with an optical spectrum analyser. As noticed in Figure 3(c), the transmitted power in the visible range decreases with agar concentration. In addition, besides the possible waveguide losses, water absorption bands were also observed in the structured fibre spectrum at 950 nm and >1200 nm^26^.

Furthermore, Figure 3(d) shows the optical loss for 2% w/v agarose fibres evaluated by the cut-back method using a HeNe laser, yielding 3.23 dB/cm (attenuation coefficient of 0.74 cm^−1^). This result is significantly lower than the 13 dB/cm reported for agarose rib waveguides^4^ but higher than the obtained for alginate-based fibres (0.45 dB/cm)^18^. Nevertheless, the lowest optical losses are expected at ~800 nm and ~1100 nm due to the improved transmittance at this wavelength, as shown in Figure 3(c).

### Sensing applications

**Figure 4:**
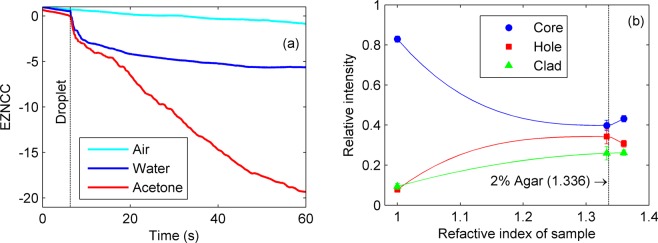
Sensing with the agarose structured fibre: (**a**) effect of surrounding fluid on the EZNCC. The sample dripping event is indicated by the vertical line. (**b**) Relative intensity values of the light transmitted by fibre core, cladding, and holes. Solid lines are guides to the eye, whereas the vertical line indicates the RI of 2% w/v agarose.

The ability of agarose to undergo structural changes under temperature, humidity, and pH variations makes the structured fibre suitable for optical sensing purposes. For example, Figure 4(a) presents the fibre response to surrounding fluids. A fluid sample (water or acetone) is dripped on the 2% w/v agar fibre excited with HeNe laser, whereas the output speckle field is monitored during 1 min by a CCD camera. Subtle variations in acquired images are quantified with the extended zero-mean normalized cross-correlation (EZNCC) coefficient, providing a sensitive metric for investigating changes in the fibre status^27^. The EZNCC signals decrease due to the fact that agarose may experience volume changes by swelling, dehydration, and syneresis depending on the fluid^28^, which affect the fibre geometry and light guiding conditions, and cause spatiotemporal variations in the speckle patterns.

An alternative to the previous sensing method is measuring the effect of fluids inserted into the air holes. A 2% w/v agarose fibre (refractive index of 1.336) is filled with air (1.000), water (1.333), and ethanol (1.361), whereas a HeNe laser excites the fibre core, and the output speckle is projected on a screen and measured by the CCD camera. Average intensities in the core, cladding, and holes are shown in Figure 4(b): as the RI difference between core and holes reduces, light confined in the core leaks through suspending bridges and is partially coupled to holes and cladding^29^. Therefore, fluid samples can be identified by comparing the relative intensities observed for each region of the projected fibre speckle field.

## Discussion

The refractive index and numerical aperture of the optical fibres can be adjusted by choosing the agarose content or adding sucrose to the solution. Moreover, mechanical strength and stability at ambient conditions are also expected to be improved for higher agarose concentrations^30,31^. On the other hand, overall transmittance is lowered with the increase of agar content, probably due to scattering by microscopic air bubbles retained during solidification. Occurrence of bubbles depends on several parameters such as mixing speed, temperature, and agarose concentration, but can be prevented by applying ultrasound treatment or increasing the settling time of the solution^4,32,33^. Another aspect is the scattering by aggregates of helical agarose molecular structures generated after gelation, leading to an increase in sample turbidity and, consequently, hindering transmittance. Although such an effect becomes more conspicuous for highly concentrated samples, it could be reduced by adding sugar to the mixture^30,31^.

For the sake of comparison, Table 1 summarizes the characteristics of hydrogel fibres and planar waveguides reported in previous works. The optical loss for the structured fibre is compatible with other agarose and alginate-based devices, but fibre dimensions must be reduced to improve light guiding and coupling conditions. On the other hand, larger cores are suitable for speckle field interrogation, as the increased number of guided modes produces a more granular and thus sensitive output speckle pattern^24^.

**Table 1:** Characteristics of hydrogel-based optical fibres and waveguides reported in the literature.

Ref.	Material	Type	Dimensions	Optical loss
This	Agarose	Structured fibre	0.64 mm core, 2.5 mm cladding (diameter)	3.23 dB/cm(633 nm)
^4^	Agarose	Rib waveguide	130 × 130 µm² (cross-section area)	13 dB/cm(633 nm)
^23^	Gelatin core, agarose cladding	Planar waveguide	2 µm–2 mm core (thickness)	N/A
^7^	Polyethylene glycol diacrylate (PEGDA)	Planar waveguide	1 × 4 mm² (cross-section area)	0.17–0.68 dB/cm(450–550 nm)
^18^	PEGDA	Planar waveguide	1.1 ×5 mm² (cross-section area)	1.7–2.8 dB/cm(450–750 nm)
^1^	PEGDA core, alginate cladding	Fibre	0.2–1 mm core, 0.4–1.2 mm cladding (diameter)	0.3 dB/cm(492 nm)
^6^	PEGDA core, alginate cladding	Fibre	0.2–2 mm core, 0.3–2.2 mm cladding (diameter)	2.6–8.3 dB/cm(532 nm)
^19^	PEGDA core, alginate cladding	Fibre	0.2–2 mm core, 0.3–2.2 mm cladding	1–6 dB/cm(532 nm)
^20^	Alginate-polyacrylamide	Fibre	0.4–1.1 mm core, 0.9–2.2 mm cladding	0.45 dB/cm(532 nm)

It must be stressed that the 3.23 dB/cm loss is still high in comparison to glass^2^ and polymer fibres^3,11,12^, and fabrication is also restricted by the mould dimensions, so it is not possible to produce kilometres of fibres like in preform drawing towers. Nevertheless, short fibre segments can be coupled to silica launching and collecting fibres to be used in laboratory setups for biochemical sensing and light delivery in which the transmission losses do not critically compromise the system response^16,17^.

Concerning the measurement of surrounding fluids, Figure 4(a), the correlation coefficient decreases continuously in case of the waveguide subjected to the air, which is associated to a gradual volume reduction by syneresis^28^, i.e., slight fibre geometry deviations affect the core modes distribution, leading to spatiotemporal changes in the speckle pattern. On the other hand, adding a water droplet yields an abrupt variation followed by a slow decrease of the EZNCC signal, whereas acetone produced a faster decay tendency. Notwithstanding the initial variation is caused mostly by mechanical disturbance, the subsequent speckle field drift can be explained by agarose swelling due to water absorption, or shrinkage related to dehydration with acetone. These results corroborate the behaviour of bulk agar samples, wherein absolute volume changes obtained by immersion in water are less noticeable than those observed for acetone^28^. Therefore, one may apply the agarose fibre as a consumable chemical sensor: regarding an *in vivo* application, for example, the optical signal decay rate due to the fibre degradation could be related to biochemical parameters in-loco for investigating diseases.

With respect to the fibre filled with air, water, and ethanol, Figure 4(b), the refractive indices difference between 2% w/v agar (1.336) and air (1.000) causes the light to be highly confined to the core, as expected. Conversely, the RI of water (1.333) is quite similar to the agar one, therefore power is leaked through the suspending agarose bridges and is partially coupled to the holes and cladding, increasing their respective relative intensity values^29^. Finally, regarding ethanol, leaked light is fairly coupled into the holes because the RI in this region (1.361) becomes greater than the surrounding agar substrate. It is worth noticing that sensitivity and measurement range can be enhanced by adjusting the composition of agarose solution (i.e., the RI value), so the optical fibre would be capable to assess a variety of substances. Furthermore, functionalization of agarose is also possible, expanding the range of detectable chemical and biological agents^34,35^. Alternatively, one may investigate the transmitted spectrum of core-propagating modes since part of the lightwave travels as evanescent fields that interact with the filled holes, like in fluorescence measurements^25^. Indeed, changing the configuration of stacked rods inside the mould is feasible for creating structures with different geometries and core sizes, which may be useful for controlling the modal distribution and designing additional holes for improving the light guidance or its interaction with measurands. Ultimately, filling the air holes with different fluids might be an interesting approach for characterizing samples based on the refractive indices mismatch, which affects the light intensity distribution.

Another promising application is using the agarose fibre simultaneously as an optical sensor and growth medium for microorganisms^4,36^. Both excitation and fluorescent lights can be guided through the structured fibre core, making it simple to evaluate the spectral response under different environmental conditions. The waveguide may be designed as a disposable sample unit containing the necessary nutrients and immobilized cells, which could be promptly coupled to the optical instrumentation (like a spectrometer or microscope) for practical analyses.

In conclusion, this paper reported the fabrication and characterization of a structured optical fibre made of agarose, in which the structured fibre geometry and the composition of the hydrogel solution can be adjusted to achieve the desired optical characteristics. Besides the prospective uses for *in vivo* imaging and light delivery, the agarose fibre may be suited for chemical measurements based on the sensitivity provided by the waveguide material and the holey structure, with potential applications for *in vivo* monitoring as the agar is biocompatible and edible. Moreover, the fibre can also be used as a substrate for cell immobilization and as a growth medium, so the biochemical properties can be assessed through the interaction between the sample and the transmitted light. In this sense, future works will be focused on optimizing the structured fibre design to minimise the transmission losses, characterizing additional fibre properties such as nonlinear refractive index and group velocity dispersion, and investigating its biochemical sensing capabilities in practical scenarios.

## Methods

### Fabrication of structured optical fibre

Food-grade agar powder is added to distilled water and heated in a hot plate (IKA R Basic KT/C). The solution is continuously agitated at ~100 °C for 2 min. Then, the temperature is lowered to ~80 °C and the sample is kept at rest for 2 min to remove air bubbles. The structured fibre mould is comprised of a 3 mm inner-diameter glass tube with six 0.5 mm diameter rods aligned with the tube axis. The rods are arranged symmetrically and fixed to the external tube using polymer holders, whereas complementary central rods are connected to the extremities to support the structure and create the fibre core. After pouring the liquid agar solution inside the mould, the tube is sealed with Teflon tape and cooled in a refrigerator until the sample becomes solid. Finally, the rods are carefully removed to form the air holes, the fibre is pushed from the mould, and the end faces are cleaved using a razor blade.

### Fibre characterization

All the tests were conducted at room temperature. The cross-section image of the optical fibre was obtained using a Nikon Eclipse E200 microscope equipped with 4×/0.1 objective. To demonstrate the guiding characteristics, light from a Newport HeNe laser (633 nm, 5 mW) was coupled to the fibre core through a 40× objective, whereas the output field was expanded by a 10× objective and acquired with a digital camera.

The refractive index was measured in a MISCO PA202 Palm Abbe refractometer with 1 × 10^−4^ RIU resolution at 589.29 nm. Samples were prepared by diluting agar powder in 50 mL of distilled water, with a further addition of food-grade sucrose into the boiled agarose solutions. The solidified materials were cut into ~1 mm side cubes and carefully placed over the refractometer detector to avoid air interfaces.

Transmission in bulk agarose samples was measured with a Hitachi U-3000 spectrophotometer (±0.3 nm accuracy within the 190 to 900 nm range) using 10 mm path length quartz cuvettes. Agar solutions with different concentrations were solidified inside the cuvettes and analysed using DI water as a reference. Regarding the output power for agarose fibre, an NKT Photonics SuperK Compact supercontinuum source was used to illuminate a 30 mm long fibre, whereas the light was collected by a multimode fibre, then measured with a Yokogawa AQ-6315 optical spectrum analyser (~0.05 nm resolution from 350 to 1750 nm).

The waveguide loss was determined by the cut-back method using the HeNe laser and an u-Eye IDS UI-2230SE-C HQ CCD camera (1024 × 768 pixels), wherein the input and output powers were calculated as the average pixel values in grayscale. The experiments were performed for 30 mm length, 2% w/v agar fibres placed on a microscope slide and cleaved in steps of ~5 mm. Finally, the loss in dB/cm was evaluated by fitting the experimental data with a linear function.

### Measurement of surrounding fluids

**Figure 5:**
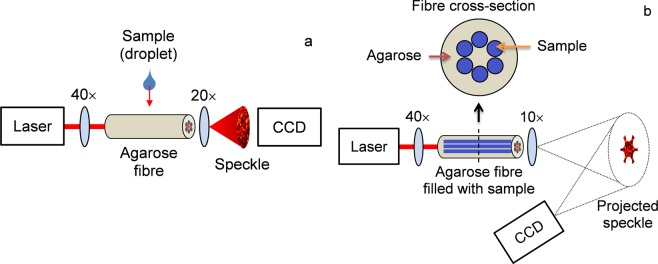
Experimental setups for fibre sensing: (**a**) measurement of surrounding fluid droplet by speckle field analysis; (**b**) assessment of fluids inserted into all the fibre holes based on the average intensity of projected speckle pattern.

Volume changes experienced by agar in response to the surrounding medium were assessed using the setup depicted in Fig. 5(a). The 2% w/v agarose fibre is inserted in a plastic tube with a small aperture for dripping the sample and to prevent the liquid from flowing into the air holes. Droplets are poured using a micropipette, which is enough for covering the exposed cladding surface without displacing the fibre inside the tube. Light from a HeNe laser is coupled to the fibre core using a 40× objective, whereas the output beam expanded by a 20× lens is detected by the CCD camera. Several propagating modes are excited because of the core dimensions, and the interference between these multiple modes produces an output speckle pattern. The intensity of a fibre speckle field projected over a *xy* plane is given by

1 $$I(x,y)=\mathop{\sum }\limits_{m=0}^{M-1}\mathop{\sum }\limits_{n=0}^{M-1}{a}_{m}{a}_{n}\exp [j({\phi }_{m}-{\phi }_{n})],$$

*M* is the number of modes, and *a*_*m*_ and *ϕ*_*m*_ are the amplitude and phase distributions of the *m*-th mode^24^. Volume changes in agar structure lead to deviations in light-guiding conditions, which affects the spatiotemporal configuration of *I*(*x*,*y*)^37^. A sensitive, non-saturating metric for speckle field monitoring is the extended zero-mean normalized cross-correlation function,

2 $${\rm{EZNCC}}={Z}_{0}\mp \left\{\frac{\iint ({I}_{0}-{\bar{I}}_{0})(I-\bar{I})dxdy}{{[\iint {({I}_{0}-{\bar{I}}_{0})}^{2}dxdy\iint {(I-\bar{I})}^{2}dxdy]}^{1/2}}-1\right\},$$

*Z*_0_ is a cumulative offset, *I*_0_ is the speckle distribution for a reference fibre status, and the overlines indicate average values^27^. The acquired data are processed by routines developed in MATLAB (Mathworks) that convert the speckle field image to grayscale, reduce it to a square 201 × 201 pixels region-of-interest, apply a 2D discrete wavelet transform (Daubechies db4) filter, and evaluate the EZNCC.

### Measurement of filling fluids

To assess liquids or gases inserted into the air holes, a 2% w/v agarose fibre core is filled with the analysed sample by immersing the fibre tip into the liquid and using a syringe attached to the other end face for drawing fluids inside the air cavities. This procedure is repeated until all holes are completely filled with liquid and no air bubbles can be observed. Subsequently, the fibre is cleaved and the core is excited with a HeNe laser, while the output beam expanded by a 10× objective is projected on a screen and captured by a CCD camera, as shown in Fig. 5(b). Finally, the normalized speckle field intensities in the core, cladding, and holes are evaluated by image processing routines implemented in MATLAB.

## Data Availability

Datasets and original images are available from the corresponding author on request.
